# Decoding the spatial logic of immune evasion in hepatocellular carcinoma: from single-cell profiling to spatial omics

**DOI:** 10.3389/fimmu.2026.1935648

**Published:** 2026-09-22

**Authors:** Naite Xi, Shan Gao, Chaoliu Dai

**Affiliations:** 1 Department of General Surgery, Shengjing Hospital of China Medical University, Shenyang, China; 2 Department of Anesthesiology, Shengjing Hospital of China Medical University, Shenyang, China

**Keywords:** hepatocellular carcinoma, immunotherapy, single-cell analysis, spatial omics, tumor microenvironment

## Abstract

Hepatocellular carcinoma remains difficult to treat because immune resistance is organized not only by malignant cell states but also by the spatial architecture of the tumor microenvironment. Single-cell RNA sequencing has clarified cellular heterogeneity in liver cancer, yet tissue dissociation removes the positional relationships that determine whether immune cells can reach and eliminate tumor nests. Spatial transcriptomic, proteomic, and multiplex imaging technologies now make it possible to map immune exclusion, stromal barriers, vascular gatekeeping, tertiary lymphoid structures, macrophage and fibroblast programs, and metabolic checkpoints within intact tumor tissue. This review integrates recent single-cell and spatial multi-omics evidence to explain how suppressive neighborhoods emerge in hepatocellular carcinoma and how they shape immunotherapy response. We discuss the mechanisms through which malignant, myeloid, lymphoid, endothelial, and stromal populations interact across spatial niches, and we examine how these maps can inform biomarker discovery, patient stratification, and rational combination therapy. We also highlight limitations in cohort design, sampling depth, analytical integration, and clinical validation that currently constrain translation. By linking cellular states to their tissue coordinates, spatial omics provides a framework for understanding why ICI succeed in some tumors but fail in others. Integrating single-cell resolution with spatial context may help convert descriptive tumor atlases into clinically actionable maps for precision immuno-oncology.

## 1 Introduction

Hepatocellular carcinoma (HCC) is the sixth most common cancer and the third leading cause of cancer-related death worldwide (1). Immune checkpoint inhibitors (ICIs) have changed the treatment landscape for advanced HCC and can improve survival compared with conventional systemic therapies such as sorafenib. However, most patients still fail to achieve durable long-term responses (2). This limited efficacy is closely linked to the tumor microenvironment (TME), which coordinates dynamic interactions among T cells, cancer-associated fibroblasts (CAFs), tumor-associated macrophages (TAMs), and the extracellular matrix (ECM) (3). The HCC TME is highly complex at cellular, molecular, and architectural levels, and this complexity can restrict immunotherapy efficacy (4). Immune states vary not only between patients but also within individual lesions. Different regions of the same tumor may display immune-excluded or immune-inflamed phenotypes (5). Consequently, single-site biopsies may fail to capture the full immune state of a tumor, complicating diagnosis, prognosis, and patient stratification (6). This narrative review focuses on recent literature connecting single-cell profiling, spatial omics, immune architecture, and translational biomarkers in hepatocellular carcinoma.

Single-cell RNA sequencing (scRNA-seq) has become central to the study of the TME (7). By profiling transcriptomes at cellular resolution, scRNA-seq has transformed the characterization of normal and diseased tissues (8). In HCC, large-scale scRNA-seq atlases of hepatitis B virus (HBV)-related disease (9), early-stage lesions (10), and recurrent or late-stage tumors (11) have revealed diverse immune and malignant cell phenotypes. A core limitation, however, is that scRNA-seq requires tissue dissociation. This process removes cells from their native topological context and obscures proximity-dependent communication circuits (12). Spatial omics complements scRNA-seq by linking molecular identity to tissue architecture. Whereas scRNA-seq resolves phenotypic states, spatial omics shows where these states occur and where immunosuppressive interactions are likely to form. This integration is particularly important for identifying spatially constrained cellular niches that may drive tumor evolution or ICI resistance (13). A comparison of dissociative single-cell profiling and spatial omics platforms is provided in **Table 1**.

**Table 1:** Comprehensive comparison of scRNA-seq and spatial omics technologies in oncology.

Technology	Representative platforms	Strengths/resolution	Oncology applications	Key limitations
Dissociative single-cell profiling	10x Chromium; Smart-seq2; BD Rhapsody	True single-cell transcriptomes; unbiased whole-transcriptome profiling; high throughput for diverse cell states.	Dissects ITH, rare tumor or immune states and lineage trajectories; infers ligand-receptor communication.	Removes spatial coordinates; dissociation-related survival bias; cannot confirm physical cell-cell contact.
Sequencing-based spatial transcriptomics	Visium (10x Genomics); Stereo-seq (BGI); Slide-seq	Whole-transcriptome mapping across large tissue areas; resolution ranges from multi-cellular spots to subcellular/nanoscale platforms.	Maps tissue-scale architecture, spatial gene modules, subclonal competition zones and tumor-stroma boundaries.	Resolution-sensitivity trade-off; multi-cellular spots require deconvolution; high sequencing cost.
Imaging-based spatial transcriptomics	Xenium (10x Genomics); CosMx SMI (NanoString); MERFISH	Subcellular molecular localization; effective cell-level resolution depends on sampling, detection, segmentation and tissue quality.	Defines cellular neighborhoods; maps proximity-based interaction patterns; resolves immune aggregates and targeted *in situ* signatures.	Limited to predesigned panels; computationally intensive segmentation; strict RNA-quality requirements.
Spatial metabolomics	MALDI-MSI; DESI-MSI	Label-free detection of metabolites, lipids and small molecules; tissue-level to multi-cellular resolution (typically 10-50 μm).	Visualizes *in situ* metabolic reprogramming, metabolite gradients, exclusion zones and nutrient competition.	Poor FFPE compatibility; no single-cell resolution; difficult integration with transcriptomics.
Spatial proteomics/high-plex IHC	PhenoCycler/CODEX; IMC; MIBI-TOF	Subcellular protein localization; direct functional-state readout; compatible with archival FFPE tissue.	Profiles protein-level cell states; defines immune-stromal barriers; supports multiplexed clinical signatures.	Lower multiplexing than transcriptomics; costly antibody validation; spectral overlap/channel constraints.

Reported molecular or optical resolution should not be interpreted as equivalent to effective biological cell resolution.

To systematically guide readers through the multidimensional landscape of the HCC microenvironment, we provide a conceptual roadmap outlining the core framework of this review (**Figure 1**). Specifically, single-cell RNA sequencing inherently loses spatial coordinates, necessitating its integration with spatial omics to accurately map cellular phenotypes onto the native tissue architecture. This integrative approach decodes the spatial logic of HCC by revealing a unique spatial conformation. These spatial and mechanistic insights drive novel clinical applications, wherein context-dependent topological biomarkers and spatially weighted models refine patient prognostication, and spatial engineering therapies physically breach barriers to restore antitumor immunity.

**Figure 1:**
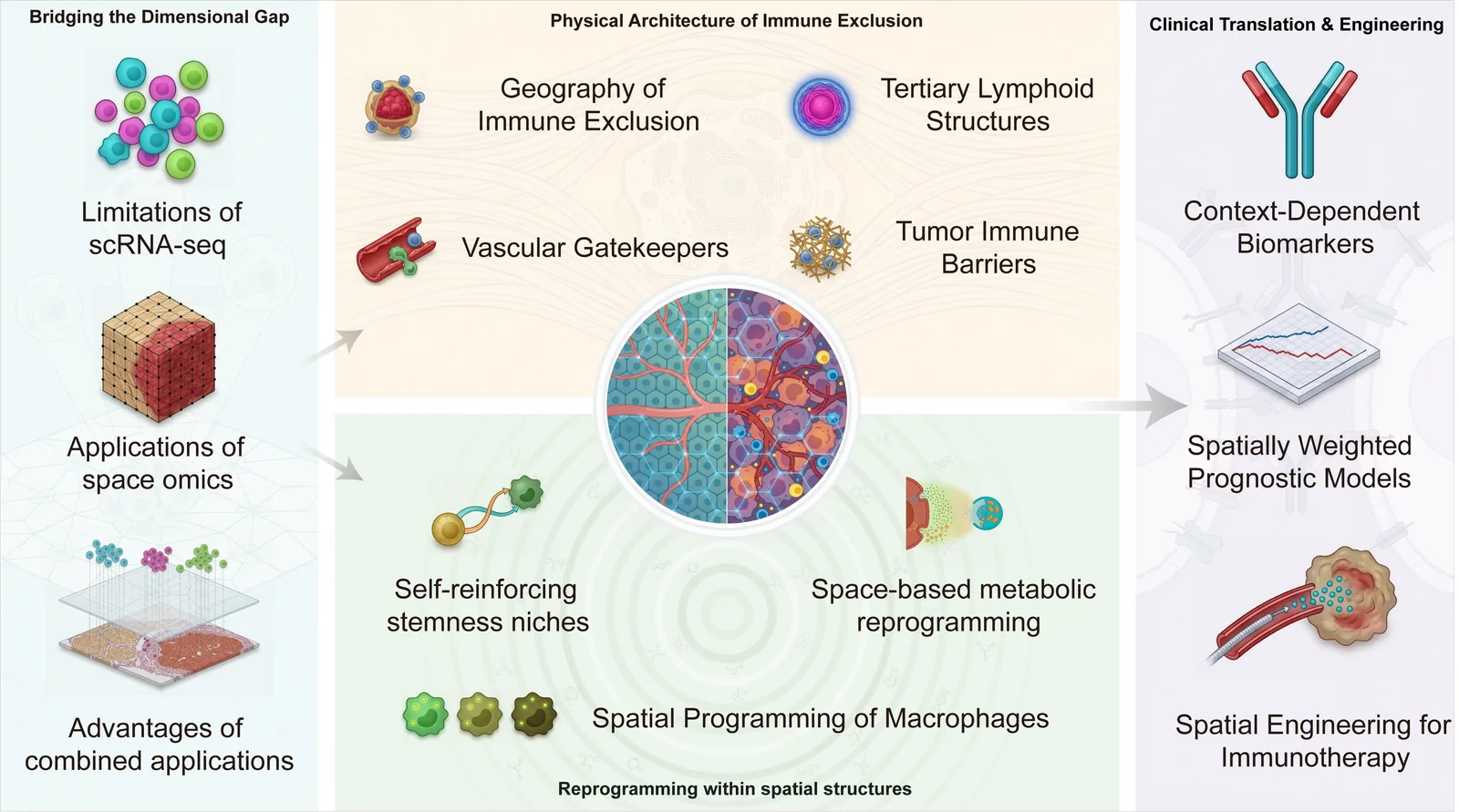
Overview of the review framework. The figure outlines the transition from the technical rationale for spatial profiling (left), to spatial mechanisms of immune evasion, including immune barriers and microenvironmental reprogramming (center), and finally to clinical translation through spatial biomarkers and spatial engineering strategies (right).

## 2 Heterogeneity and immune landscape in HCC

Before considering the spatial topology of HCC, it is necessary to define the cellular and functional landscape revealed by single-cell transcriptomics. scRNA-seq has clarified the transcriptomic plasticity of malignant hepatocytes by profiling individual cells rather than bulk averages. This approach can resolve intratumoral heterogeneity (ITH) and stemness-associated programs linked to recurrence. For example, malignant subpopulations with distinct metabolic programs have been associated with aberrant lipid metabolism and poor prognosis (14). EpCAM+ subpopulations with stem-like properties have also been implicated in self-renewal and tumorigenicity (15). Comparative studies of primary and recurrent tumors further suggest that primary lesions are often enriched for active cell-cycle subclones, whereas recurrent tumors show more immune-evasive features (16). Together, these datasets have refined the molecular classification of tumor cells and linked specific subclones to immunosuppressive niches.

scRNA-seq has also reshaped the understanding of immune and stromal heterogeneity in the HCC TME. CD8+ T cells can progress toward terminal exhaustion, with impaired cytotoxic T lymphocytes co-expressing checkpoint genes such as PDCD1, HAVCR2, LAG3, and LAYN (17). In the myeloid compartment, the field has moved beyond the M1/M2 framework because this binary scheme incompletely captures the continuous, plastic, and context-dependent macrophage programs observed in human HCC. The populations discussed below, including TREM2+, SPP1+, and phosphorylation-defined TAM states, should therefore be interpreted as partially overlapping functional states shaped by local metabolic, stromal, and inflammatory cues, rather than as mutually exclusive lineages (18–20).

The central limitation of scRNA-seq is therefore not cellular resolution but spatial absence. Dissociation separates cell identity from tissue architecture, creating a gap between the presence of a cell state and its function within a local niche. For example, ligand-receptor analysis may infer a pro-tumorigenic axis between SPP1+ TAMs and CD44+ tumor cells (21), but transcriptomic co-expression alone cannot show whether these cells are sufficiently co-localized to signal *in situ*. Similarly, scRNA-seq can identify myofibroblastic CAFs (myCAFs) as drivers of matrix remodeling (22), but it cannot visualize the physical barriers that prevent CD8+ T cells from reaching tumor islets. Tissue homogenization also destroys niches such as the immune-suppressed core and immune-active invasive margin, which may be essential for interpreting HCC heterogeneity (23, 24). Thus, spatial omics is needed to test inferred communication circuits within their native architectural context.

This spatial gap also limits the clinical interpretation of transcriptomic states. Heterogeneity in HCC is not only compositional but also architectural. Oncofetal reprogramming (25) and drug-resistant clones (26) may be shaped by local microenvironmental context. Single-cell transcriptomics alone cannot determine whether immune-excluded patterns are linked to poor survival or specific genomic alterations (25, 27). Without mapping high-dimensional cell states to tissue structure, key spatial cues for treatment failure and spatially restricted therapeutic targets may be missed (28, 29). The transition from dissociation-based profiling to spatially resolved analysis is therefore a critical step toward connecting cellular identity with clinical behavior.

## 3 Decoding the spatial architecture of the microenvironment

Spatial omics helps bridge this dimensional gap by mapping cellular identities back onto tissue architecture. These technologies establish spatial coordinate systems that can be integrated with transcriptomic, proteomic, and metabolomic information. When combined with scRNA-seq, spatial omics preserves both phenotypic resolution and positional context. This integrated strategy is increasingly used to define how cellular components of the HCC microenvironment are organized *in situ*.

### 3.1 Spatial zonation and vascular gatekeepers

As comprehensively illustrated in **Figure 2**, examining the spatial logic of the HCC TME reveals how the macro-architectural arrangement orchestrates processes driving immune evasion, invasion, and therapeutic resistance. This spatial evolution encompasses distinct trajectories: the macroscopic transition from a functional antitumor invasive margin to an immunosuppressive core, the selective gating by tumor-associated vasculature, and the structural divergence of tertiary lymphoid structures (TLSs).

**Figure 2:**
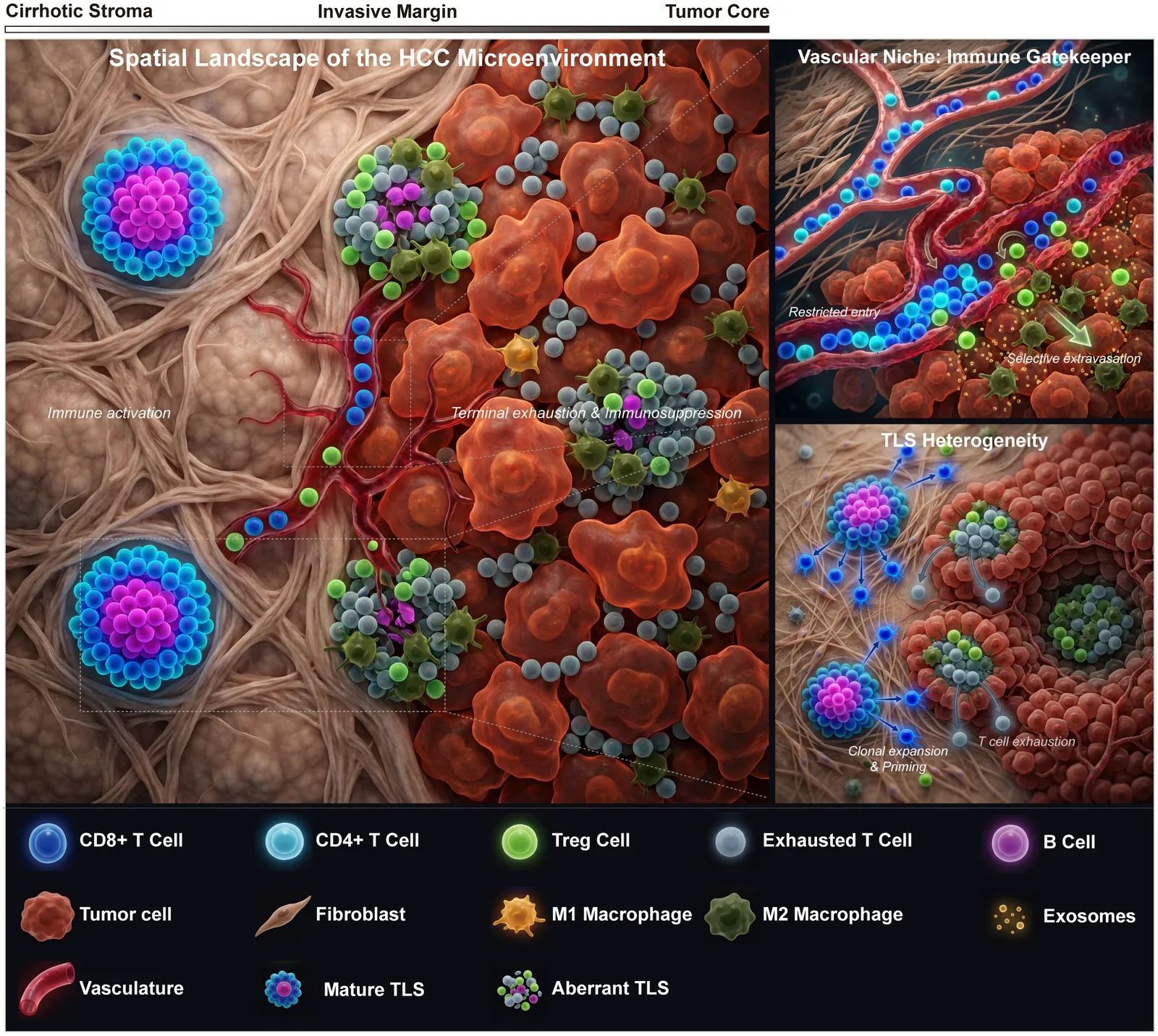
Spatial evolution, vascular gating, and TLS heterogeneity in HCC. The HCC microenvironment shows a structural and immunological gradient from a fibroblast-rich cirrhotic stroma with functional antitumor immune cells at the invasive margin to a dense, hypoxic tumor core. The core is often enriched with immunosuppressive macrophages, regulatory T cells (Tregs), and exhausted T cells. Tumor-associated vasculature further shapes this localized immunosuppression by restricting effector T-cell transmigration while facilitating suppressive cell trafficking. TLS localization and maturation also influence T-cell trajectories. Mature TLSs in peritumoral stroma support organized B-cell and T-cell zones, whereas aberrant TLSs in hypoxic tumor regions may be infiltrated by suppressive populations and fail to sustain effective immune activation. HCC, hepatocellular carcinoma; TLS, tertiary lymphoid structure; Treg, regulatory T cell.

#### 3.1.1 Geography of immune exclusion

A major insight from spatial profiling is the existence of core-margin zonation, which reflects the interplay between host surveillance and tumor adaptation. Immunosuppression is not randomly distributed across the tumor. Instead, recent multi-omics analyses suggest that it can emerge at the interface between the tumor and the surrounding cirrhotic liver. This interface may function less as a conduit for immune attack than as a site of immune regulation. The cirrhosis-damaged peritumoral area resembles a tissue repair zone, where cytotoxic CD8+ T cells, M2-like TAMs, and regulatory CD4+ T-cell hubs contribute to a tumor-permissive environment (30). This positioning may allow HCC to exploit the liver’s intrinsic tolerance mechanisms, which normally limit excessive inflammation during fibrosis.

The transition from the regulatory margin to the tumor core reveals a second layer of spatial organization, especially in metabolic dysfunction-associated steatohepatitis (MASH)-related HCC. Spatial profiling shows a decline in functional immune populations, including PD-1+ CD8+ T cells and inflammatory macrophages, from the periphery toward the core (31). In the core, impaired infiltration is compounded by hypoxia, nutrient deprivation, and metabolic exhaustion. These conditions can produce an immune-desert-like state in which T cells recognize antigen but fail to sustain cytotoxic function (30, 31). During recurrence, the margin may become further rewired toward immune suppression. High-resolution mapping has identified PD-L1+ CD103+ dendritic cells at recurrent tumor boundaries, where they are associated with recruitment of regulatory T cells (Tregs) and exhausted T cells (32). Concurrent stromal remodeling, including reduced collagen density and disordered fiber orientation, may facilitate invasion while strengthening immune suppression (32). Together, these observations support a core-margin model in which the margin serves as a regulatory trap and the core acts as a hypoxic, exclusionary niche.

#### 3.1.2 Vascular niche

This spatial logic is reinforced by tumor vasculature, which functions not only as a nutrient supply route but also as an immune gatekeeper. Endothelial-TME interactions appear highly specialized and may be shaped by malignant subclones that co-opt angiogenesis. Angiogenesis is therefore not uniform across the tumor. For example, SORT1+ tumor subclones enriched in the tumor core can promote local angiogenesis through oncogenic signaling to neighboring endothelial cells (33). Similarly, GOLM1 (GP73)+ tumor cells that produce VEGFA and CXCL5 physically co-localize with angiogenic endothelial subsets EC1 and EC5, whereas homeostatic endothelial cells remain more distant (34). These findings suggest that aggressive tumor subclones may construct vascular niches that support oxygen supply, dissemination, and local immunosuppression.

The immunosuppressive role of vascular niches is particularly evident in microvascular invasion (MVI) and portal vein tumor thrombus (PVTT). Spatial analyses indicate that MVI+ regions are not only anatomically invasive but also biologically distinct metastatic niches. These regions contain cycling T cells, LAMP3+ dendritic cells, and TREM2+ TAMs that may act in concert (35). Vascular endothelial cells can also release CD63+ exosomes that are taken up by neighboring immune cells, promoting broader immunosuppressive programs (30). In addition, pathological vasculature may regulate immune-cell extravasation by permitting Treg trafficking while limiting effector T-cell entry (36, 37). Thus, tumor vasculature may selectively filter immune infiltration and reinforce spatially restricted suppression.

#### 3.1.3 Tertiary lymphoid structures

Beyond vascular barriers and suppressive gradients, the immune system may attempt to organize local defense through TLSs. Spatial omics, however, shows that the function of TLSs depends strongly on their maturation and location. Mature TLSs contain organized B-cell germinal centers marked by MKI67, IGHD, and BCL6, together with adjacent T-cell zones that support antibody generation and memory responses (38). In HCC, some TLSs appear structurally aberrant and are infiltrated by suppressive populations such as FOLR2+ Tregs and M2-like TAMs (38, 39). TDO2-expressing malignant cells may further impair TLS maturation by creating a tryptophan-depleted niche (38). However, the specific contribution of FOLR2+ regulatory populations requires direct HCC-focused spatial and functional validation. Evidence from other tumor types is discussed here only as a mechanistic hypothesis and should not be interpreted as HCC-specific proof.

### 3.2 Spatially orchestrated microenvironmental reprogramming

After defining the spatial architecture of HCC, the next question is how these boundaries reprogram the cells within them. Integrating spatial omics with single-cell resolution has moved the field beyond static cellular inventories. The question has shifted from who is present to how cells communicate within defined neighborhoods. Recent studies have begun to resolve the coordinates of ligand-receptor interactions, post-translational modifications, and metabolic fluxes. These data suggest that immune evasion is maintained by spatially organized reprogramming networks rather than by any single cell type.

#### 3.2.1 Spatial programming of macrophage identity

Macrophages sit at the center of this regulatory network. Spatial omics has revealed macrophage states shaped by local environmental cues and metabolic sensing. One example is the interaction between tumor subclones and TREM2+ TAMs. In the tumor core, SQLE is upregulated in metabolic-prototype HCC cells, leading to excess oxidized low-density lipoprotein (oxLDL) production (19). oxLDL can engage macrophages through the TREM2-SYK-CEBPα axis, promoting an immunosuppressive state associated with reduced CD8+ T-cell penetration (19). This mechanism links tumor metabolic reprogramming to immune exclusion within a defined spatial niche. Chemokine gradients add another layer. High CCL15 expression in the tumor core recruits and polarizes CD163+ M2-like macrophages, creating a dense immunosuppressive barrier that can repel effector cells (40). Spatial profiling also suggests that macrophages amplify Treg function. VIM-expressing macrophages co-localize with Tregs and secrete IL-1β, which activates Tregs to release IL-10 and propagate suppressive signaling (41).

Spatial proteomics further indicates that macrophage function may be tuned by niche-specific post-translational states. One study mapped phosphorylation of HSP27, STAT1, and TRIM28 in M2-like macrophages and identified four spatial subtypes (M2-P0 to M2-P3) (42). M2-P0 macrophages in peritumoral tissue lacked these phosphorylation marks and appeared more immune active. As macrophages infiltrated deeper into the tumor, phosphorylation states increased progressively. M2-P1 and M2-P2 macrophages at the tumor periphery were associated with early CD8+ T-cell exhaustion. In the tumor core, M2-P3 macrophages showed a tri-phosphorylated state and high expression of PD-L1, CTLA4, and TIM3. Their proximity to hepatocytes was also associated with PD-L1 and CD47 upregulation in tumor cells, forming a local suppressive synapse (42). This gradient suggests that the TME can reprogram infiltrating macrophages through spatially defined post-translational cascades.

Functional perturbation studies support this macrophage-centered spatial model. In orthotopic and spontaneous mouse HCC models, cancer-cell Sqle depletion reduced oxLDL production, TREM2+ macrophage infiltration, and immune dysfunction, while improving CD8+ T-cell activation and adding to anti-PD-1 efficacy (19). A complementary macrophage-coated tumor cluster (MCTC) model showed that tumor-derived M2BP promoted Mac-2+ macrophage adhesion around Hepa1–6 tumor cells, excluded intratumoral CD8+ T cells, and increased invasion. The Mac-2 antagonist GB1107 disrupted MCTC formation and enhanced anti-PD-1 activity in C57BL/6 mice (43).

#### 3.2.2 Fibroblasts as architects of matrix-immune interactions

Spatial omics has also reframed CAFs as active architects of immune niches rather than passive stromal scaffolds. CAFs can support tumor progression or, in specific contexts, promote antitumor immunity. In suppressive niches, CAFs and tumor cells form feedback loops. For example, S100A9+ tumor cells release SPP1, which induces neighboring fibroblasts to acquire a FAP+ phenotype. These CAFs then release CCN2 (also known as CTGF), increasing SMAD3 phosphorylation in tumor cells and promoting epithelial-mesenchymal transition and metastasis (44). This epithelial-stromal loop may generate localized regions of high aggressiveness. In contrast, FMO2+ CAFs provide a treatment-specific example in which a spatially defined state has been linked to functional validation. Integrated single-cell, spatial-transcriptomic, and patient-response analyses identified FMO2+ CAFs as enriched in anti-PD-1 responders. Coculture and orthotopic HCC models then showed that FMO2 promotes CCL19 expression, TLS formation, and CD8+ T-cell/M1-like macrophage infiltration; CCL19 treatment potentiated anti-PD-1 efficacy (45). These data support the CCL19-CCR7 axis as a mechanistically tested candidate, rather than a spatial association alone.

#### 3.2.3 Bidirectional programming of cancer stem cells and the TME

Cancer stem cell (CSC)-like populations may also participate in bidirectional niche programming. Their function appears to change with spatial context. In early lesions, EpCAM+ and PROM1+ CSC-like populations act mainly as proliferative units with limited immune interaction (46). At invasive fronts, however, CD24+ and ICAM1+ stem-like subpopulations can engage immune pathways. Through ICAM1 signaling, these cells promote M2-like macrophage polarization and T-cell exhaustion (46). In minimal residual disease, KRT19+ and EpCAM+ stem-like cells are found in direct contact with PD-L1+ M2-like macrophages. These macrophages release TGF-β1 to maintain tumor-cell stemness and dormancy, while tumor cells reinforce macrophage immunosuppression (47). PROM1+ CSC nests in the tumor core can also overlap with Treg clusters and CD47+ cells, creating a protected niche (48). Additional myeloid and stromal interactions may support this pool. CSC-derived CCL3 and CCL5 recruit CD168+ macrophages to the tumor margin, where macrophage-derived CXCL12 and TGF-β1 promote TOP2A expression in tumor cells (49). COL1A2- and CCN2-expressing CAFs also localize with stem-like nests and activate JAG1-Notch and TGF-β1/TGF-βR1 signaling (50).

Functional evidence for this axis is strongest in HCC minimal residual disease. In transgenic and syngeneic orthotopic models, PD-L1+ M2-like macrophages spatially interacted with stem-like residual tumor cells and supplied TGF-β1. Combined TGF-βR1 and PD-L1 blockade reduced suppressive macrophages, recruited activated CD8+ T cells, eliminated residual stem-like tumor cells, and prevented recurrence (47). In an early hepatocarcinogenesis model, MEF2D+ cancer precursors also reprogrammed liver-resident macrophages through NRP1-dependent Kupffer-cell state conversion, and NRP1 inhibition reduced tumor initiation in mice (51). The latter study supports malignant-cell-to-macrophage niche programming, but it addresses tumor initiation rather than established ICI resistance.

Taken together, these studies suggest that spatial immune evasion in HCC emerges from partially overlapping cell axes rather than from a single suppressive population. Malignant-cell states can recruit or reprogram macrophages, macrophages can cooperate with CAFs to form immune-exclusion barriers, and macrophage-derived signals can sustain stem-like residual tumor cells. However, no single HCC model has yet perturbed malignant subclones, macrophages, CAFs, and CSC-like cells simultaneously. The integrated network should therefore be presented as a convergent, experimentally supported model with remaining causal links to be tested.

### 3.3 CAF-based immune barriers

Regulatory signaling networks shape cellular function, but antitumor immunity also depends on physical access. Spatial omics has shown that HCC is not a homogeneous mixture of cells. Instead, immune cells and malignant cells can be separated by spatial exclusion zones. This creates a paradox in tumors that appear immunologically hot because of high overall T-cell abundance (52). Effector cells may still remain trapped in stromal regions and fail to contact malignant nests. Immune exclusion is therefore not simply a byproduct of fibrosis. It reflects an active architectural program driven by stromal and innate immune interactions.

In cirrhosis, a major precursor state for HCC, myCAFs form a structural backbone for immune exclusion (53). Activated hepatic stellate cells (HSCs) contribute to scarring and create tumor-permissive niches. Stromal desmoplasia is associated with poor prognosis and may act as a selective sieve, limiting cytotoxic lymphocyte entry while permitting suppressive signals (54). Spatial omics has clarified that collagen deposition alone does not explain this barrier. Instead, spatial and functional studies suggest that CAF-centered signaling networks contribute to the establishment and maintenance of this border (55–58).

The crosstalk between SPP1+ TAMs and CAFs is a central axis of barrier formation. Recent studies describe a tumor immune barrier (TIB) at the tumor border. As shown in **Figure 3**, the TIB can physically and metabolically separate effector T cells from the malignant core. Hypoxia at the tumor margin induces macrophages to upregulate SPP1. These SPP1+ TAMs then activate adjacent CAFs and promote ECM remodeling, strengthening resistance to immune infiltration (55). This SPP1-dependent program involves TAM-derived metalloproteinases (MMP9, MMP12, and MMP7) and signaling through CD44 or integrins on stromal cells, which reorganize the ECM into a dense mesh (33). SPP1 can also drive quiescent HSCs toward activated CAF states (56), sustaining the supply of barrier-forming cells. Clinically, higher TIB density has been associated with resistance to ICI (55).

**Figure 3:**
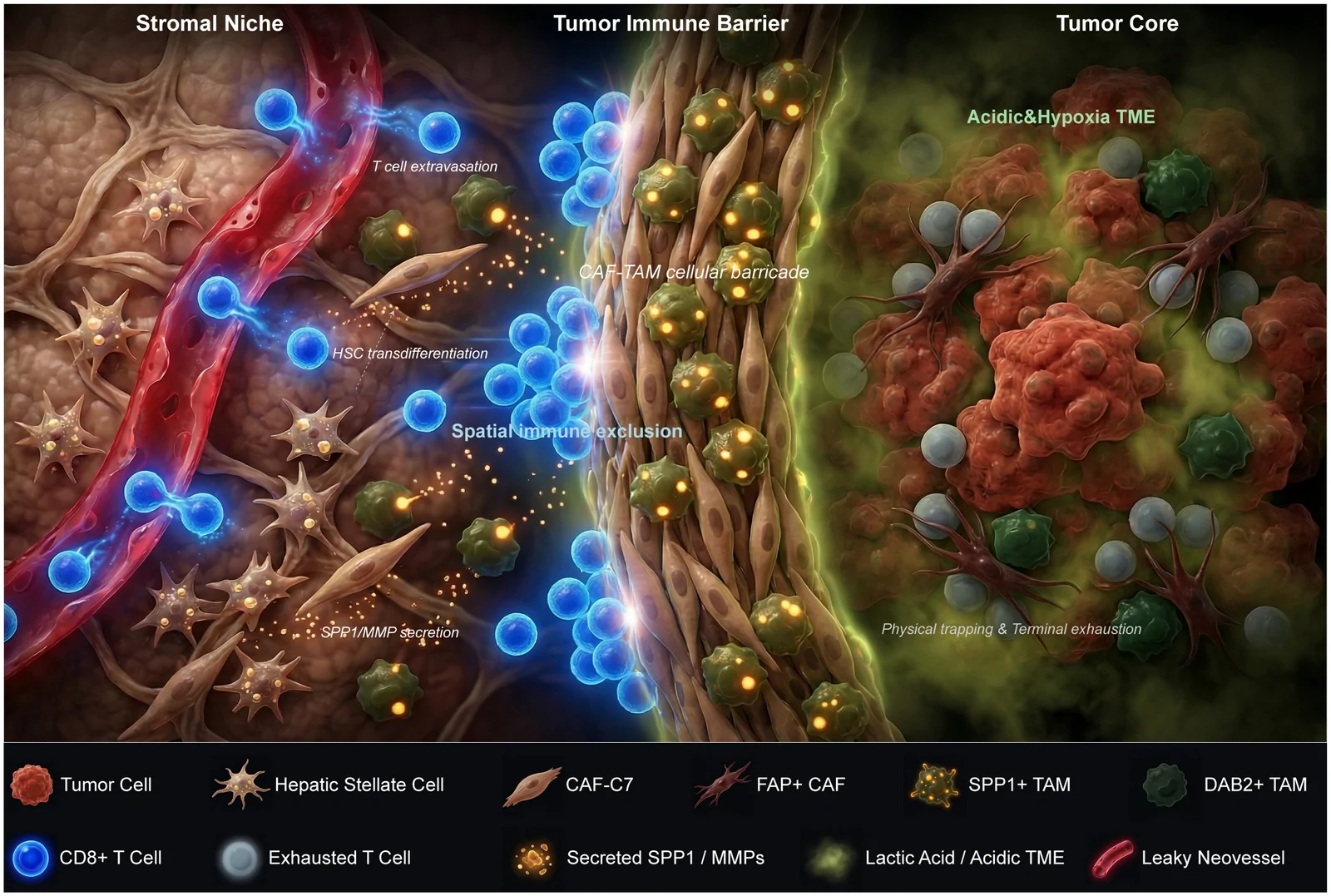
Dynamic assembly and spatial function of the CAF-mediated tumor immune barrier in HCC. At the invasive margin, leaky neovessels permit active CD8+ T cells to enter stromal regions. At the same time, hypoxia drives SPP1+ TAMs to secrete matrix-remodeling enzymes and SPP1, promoting the transdifferentiation of quiescent HSCs into active CAF lineages. CAFs and SPP1+ TAMs then assemble an interlocking cellular and matrix barrier that traps cytotoxic T cells in the stroma and limits their access to malignant nests. Within the tumor core, lactic acid accumulation and FAP+ CAF interactions with DAB2+ TAMs may further promote T-cell dysfunction and exhaustion. HCC, hepatocellular carcinoma; CAF, cancer-associated fibroblast; TAM, tumor-associated macrophage; HSC, hepatic stellate cell; SPP1, secreted phosphoprotein 1.

Functional experiments further support a causal role for this macrophage-CAF barrier. Blocking SPP1 or deleting Spp1 selectively in macrophages reduced CAF accumulation, increased cytotoxic T-cell infiltration, and sensitized mouse liver tumors to anti-PD-1 therapy (55). This evidence strengthens the interpretation that SPP1+ macrophages and CAFs form an active tumor immune barrier rather than merely co-localizing at the tumor border.

The architecture of these barriers is heterogeneous because different CAF subsets perform distinct spatial functions. High-resolution mapping has distinguished FAP+ CAFs, which colonize the tumor core, from CAF-C7 cells, which populate the boundary (56). FAP+ CAFs interact with PD-1+ CD8+ T cells and may contribute to T-cell dysfunction inside tumor nests. CAF-C7 cells interact with SPP1+ macrophages and form a peripheral barrier. This organization highlights the double-edged nature of stromal barriers. Although they exclude immune cells, they may also physically constrain tumor invasion, which could explain why dense barriers are associated with better overall survival in some cohorts (56). Other CAF lineages contribute through cytokine signaling. POSTN+ CAFs activate macrophages through the IL-6/STAT3 axis, induce SPP1 expression, and establish immune-exclusion zones enriched in ICI non-responders (59, 60). MMP11+ CAFs may further cooperate with SPP1+ macrophages to remodel matrix and consolidate exclusion (61).

POSTN+ CAFs provide another experimentally tested barrier-forming axis. In an orthotopic HCC model, POSTN knockdown reduced immune exclusion and enhanced anti-PD-1 efficacy (60). By contrast, FMO2+ CAFs promote CCL19-dependent TLS formation and antitumor immune infiltration (45), underscoring that CAF states can either reinforce or relieve immune suppression depending on their spatial program.

Spatial omics has identified multicellular hubs, termed tumor-immune-CAF interfaces, that can function as physical and metabolic barriers. At the invasive leading edge, myCAFs communicate with TAMs through lactic acid secretion, creating an acidic microenvironment that supports invasion and suppresses T-cell activity (57). These CAFs may also influence TLS formation and metabolism, thereby weakening local immune organization. FAP+ CAFs engage DAB2+ macrophages through TGF-β, PDGF, and adrenomedullin signaling (58). In this setting, FAP+ CAFs may act as integration points that limit T-cell migration (58). Overall, CAF subtypes cooperate with neighboring immune and malignant cells to build spatial barriers that reduce effective tumor rejection.

### 3.4 Metabolic architecture and spatially restricted checkpoints

Genomic and proteomic markers help define cellular identity, but the functional state of the HCC microenvironment is also shaped by metabolic architecture. Spatial metabolomics suggests that metabolic enzymes and metabolites can act as spatially restricted checkpoints. These checkpoints enforce immune exclusion through local chemical gradients.

#### 3.4.1 Lipid-lactate crosstalk at the tumor-stromal interface

Tumor cells in the core can increase expression of lipid-handling genes such as EIF3F (62) and SQLE (19), creating lipid-rich niches associated with CD8+ T-cell exclusion. Spatial omics has refined this model by identifying a tumor-immune-CAF interface in which metabolic reprogramming extends beyond malignant cells. CAFs in this zone secrete high levels of lactate and long-chain polyunsaturated fatty acids (PUFAs) (57). These metabolites are taken up by adjacent macrophages and promote M2-like immunosuppressive polarization (57). This stromal lactate-PUFA flux complements the SQLE-oxLDL axis (19) and FABP5-mediated fatty-acid metabolism in malignant clones (63). Together, these pathways may form a metabolic shield that separates effector T cells from tumor nests.

#### 3.4.2 Nitrogen recycling and the ammonia-polyamine axis

Spatial control of nitrogen metabolism adds another layer of immune evasion. Transcriptomic studies identified ODC1, the rate-limiting enzyme in polyamine synthesis, as a potential driver of the polyamine shield in PVTT tissues (64). TDO2+ cells can also deplete tryptophan and impair TLS maturation (38). Spatial metabolomics has expanded this view by revealing an ammonia-centered nitrogen-recycling mechanism. Ammonia, previously regarded mainly as a toxic waste product, can accumulate in the tumor core and support Treg expansion (65). In this high-ammonia niche, Tregs upregulate urea-cycle enzymes to reassimilate ammonia into amino acids, whereas effector T cells are more vulnerable to toxicity (65). These findings suggest a dual metabolic architecture in which polyamine accumulation and ammonia recycling selectively reinforce suppressive immune populations.

#### 3.4.3 Spatial rewiring of glucose metabolism

Glucose metabolism is another spatially organized feature of cancer. In the liver, physiological metabolic zonation separates glycolysis and gluconeogenesis along the porto-central axis to maintain systemic glucose homeostasis. During HCC development, this zonation is disrupted. Spatial multi-omics supports regional metabolic heterogeneity, including tumor-immune-CAF interfaces enriched for glycolysis- and lactate-associated programs and altered regional glucose-utilization programs (57, 66). At present, these observations establish spatial association rather than causation. Whether perturbing a defined glucose or lactate program restores effector-cell function or reduces invasion in HCC should be tested in spatially resolved functional models (67).

Together, these observations suggest that physical barriers, immune exclusion, and metabolic remodeling are not independent processes. Spatially rewired metabolism can reinforce the same suppressive architecture created by stromal and immune cells. The HCC TME should therefore be viewed as an integrated physical, immunological, and metabolic system.

## 4 Clinical translation of integrated spatial omics

Integrating single-cell gene-expression data with spatial distribution provides a framework for linking HCC heterogeneity to clinical outcomes. Spatial analysis can relate cell neighborhoods to survival, recurrence, and treatment response. The clinical value of a candidate spatial feature, however, should be assessed at three linked levels: spatial association, functional perturbation, and clinical association in a regimen-defined cohort. This framework moves beyond cellular abundance while avoiding the assumption that co-localization alone establishes a treatment mechanism.

### 4.1 Context-dependent biomarkers in a spatial context

Tumor biomarkers have long supported personalized treatment (68), but spatial omics shows that biomarker function may depend on both cellular origin and location. CCR7 illustrates this spatial duality. In malignant hepatocytes, CCR7 can promote aggressive behavior through AKT/ERK signaling (69). In stromal or immune compartments, however, CCL21-CCR7 signaling can support TLS formation (69). Bulk sequencing cannot easily reconcile these opposing roles, whereas spatial resolution can separate them by cellular and architectural context.

However, spatial context should be viewed as an added layer of biomarker interpretation rather than an automatic replacement for bulk sequencing or conventional markers. Its incremental predictive value should be tested in head-to-head models that compare spatial features with tumor stage, etiology, serum markers, bulk transcriptomic signatures, and established immunotherapy biomarkers.

Other biomarkers also acquire meaning through spatial context. PRKDC is enriched in the tumor core, where it marks hotspots of genomic instability related to error-prone DNA repair (70). TKT shows gradient expression that peaks at the invasive front and may support nucleotide synthesis (71). Junctional markers such as ADAM10 localize to the tumor-stroma boundary, where SPP1 signaling contributes to a T-cell exhaustion barrier (72). HMGB2 has likewise been reframed from a proliferation marker to a potential driver of metastatic niches in the tumor core (73).

Metabolic enzymes may function as spatially restricted checkpoints. G6PD expression forms dense metabolic islands in the tumor core and is negatively correlated with CD8+ T-cell infiltration (74). Mitochondrial-stress markers such as UQCRH and NDRG1 intersect with this glycolytic barrier. UQCRH is enriched in tumor vascular endothelium and may impair antigen presentation through reactive oxygen species (ROS) accumulation (75). NDRG1+ macrophages are enriched in the tumor core and can spatially exclude CD8+ T cells through STAT3/NF-κB signaling (76). Macrophage-specific MGAT1 expression decreases toward the tumor core, which may weaken immune function, whereas MGAT1-high macrophages are associated with improved immunotherapy response (77).

These findings indicate that the prognostic value of a biomarker is not purely intrinsic; it is also topological. Spatial omics allows biomarkers to be interpreted according to their neighborhood, such as the metabolic core or invasive margin, rather than by abundance alone. **Table 2** summarizes spatially contextualized biomarkers, cellular subpopulations, metabolic checkpoints, and their reported clinical implications, together with the available HCC cohort/sample context and evidence level. The accompanying evidence note distinguishes spatial association, cross-modal inference, functional validation, and clinical association so that candidate mechanisms are not interpreted as equally established.

**Table 2:** Spatially contextualized biomarkers, metabolic checkpoints, evidence context, and architectural programming in the HCC microenvironment.

Spatial coordinate/zone	Biomarker, metabolite, or cell subtype	Cellular origin	Primary mechanism/pathway	Spatial & functional consequence	Clinical/prognostic significance and evidence context	Ref
The Stromal & Peritumoral Compartment	Mature pTLS	B & T cells	CXCL9/10 chemokine axis	Act as functional immune command centers; actively recruit CTLs and plasma cells	A high pTLS signature strongly associates with recurrence-free survival **Evidence:** HCC TLS/outcome cohorts; etiology NR; SA+CA.	(38, 95)
	CCR7	Stromal cells	CCL21-CCR7 signaling axis	Drives the nucleation of mature TLS and immune cell recruitment	Favorable outcome **Evidence:** HCC stromal/TLS studies; etiology NR; SA.	(69)
	FMO2+ CAFs	Pro-immune CAFs	CCL19-CCR7 axis engagement	Act as immune gatekeepers; spatially cluster to recruit CD8+ T cells	Enriched in immunotherapy-responsive patients **Evidence:** HCC anti-PD-1 cohorts; coculture/orthotopic models; FV+CA.	(45)
	POSTN & MMP11	POSTN+/MMP11+ CAFs	IL-6/STAT3 signaling; enzymatic ECM remodeling	Activate macrophages to express SPP1; reinforce the dense immune-exclusion phenotype	ICI non-response; marked immune exclusion **Evidence:** HCC ICI spatial cohorts; POSTN perturbation model; FV+CA.	(59–61)
	MGAT1	TAMs	Macrophage glycosylation programming	Sustains antitumor immune function	Positive predictor of ICI response **Evidence:** HCC scRNA-seq/spatial data; etiology NR; CMI+CA.	(77)
	GZMK+ PD-1+ CD8+ T cells	Effector T cells	Intermediate exhaustion/activation post-HAIC	Emerge in neo-TLS and spread to the tumor bed to execute antitumor responses.	Indicates successful spatial engineering and a favorable outcome **Evidence:** post-HAIC HCC samples; treatment-linked SA.	(83)
The Invasive Margin & Tumor-Immune-CAF Interface	CAF-C7/myCAFs	Hepatic Stellate Cells	CD44/Integrin engagement with SPP1+ TAMs; lactic acid	Construct the physical ECM dam; block CD8+ T-cell entry while constraining tumor invasion	Dense TIB predicts ICI resistance **Evidence:** resected/cirrhotic HCC; CAF-TAM studies; etiology NR; SA+FV.	(53, 56, 57)
	SPP1	TAMs	Secretion of MMP9/12/7; paracrine crosstalk	Drives HSC transdifferentiation; orchestrates intensive ECM remodeling at borders	Correlates with immune-barrier density and poor outcome **Evidence:** HCC tumor-border TIB; macrophage Spp1 perturbation; SA+FV.	(55, 56)
	PUFAs	CAFs	Intercellular metabolic gradient secretion	Polarize adjacent macrophages toward M2-like immunosuppressive phenotypes	Orthogonal validation of the stromal metabolic shield **Evidence:** HCC tumor-immune-CAF interface; etiology NR; spatial metabolomics/CMI.	(57)
	FABP5	Malignant clones	Fatty acid metabolism	Synergizes with stromal lipid flux to chemically segregate effector T cells	Reinforces physical and chemical immune exclusion **Evidence:** human HCC metabolic studies; CMI.	(63)
	DDR1/TGF-β	α-SMA+ CAFs/Activated HSCs	Active signaling within the fibrotic niche	Actively repel infiltrating lymphocytes from the margin	Maintains a structural ceiling on therapeutic efficacy **Evidence:** HSC/HCC fibrosis models and stromal studies; FV, stage caveat.	(56, 85)
	NETs & IL-17	Neutrophils/Microenvironment	Neutrophil extracellular traps	Drive Treg proliferation at the physical barrier	Limits the efficacy of immunotherapies **Evidence:** NASH-HCC and human HCC immune studies; FV/CA; therapeutic extrapolation cautious.	(87, 88)
	CD24+/ICAM1+ CSCs	Cancer Stem Cells	ICAM1 signaling pathway	Induce M2-like macrophage polarization and T-cell exhaustion at metastatic fronts.	Drives invasive potential and immune suppression **Evidence:** HCC CSC/TME studies; etiology NR; SA+FV inference.	(46)
	PD-L1+ CD103+ DCs	Dendritic cells	Regulatory networking at recurrent boundaries	Facilitate robust recruitment of Tregs and exhausted T cells	Promotes rapid metastasis in recurrent HCC **Evidence:** recurrent HCC boundary samples; SA.	(32)
	TKT	HCC Tumor cells	Nucleotide synthesis	Provides metabolic support for outward expansion	Aggressive tumor growth **Evidence:** HCC spatial/metabolic studies; CMI.	(71)
	ADAM10	Tumor-stroma boundary cells	Junctional SPP1 signaling activation	Creates a physical and functional T-cell exhaustion barrier	Resistance to immune infiltration **Evidence:** HCC tumor-stroma boundary studies; SA.	(72)
The Deep Tumor Core & Metabolic Islands	SQLE	Metabolic Prototype cells	Reprogramming of lipid metabolism	Generates a lipid-rich niche; maintains TAMs in a suppressive state via TREM2-SYK-CEBPα.	Prevents CD8+ T cells from penetrating the core **Evidence:** HCC metabolic archetypes; orthotopic/spontaneous models; FV.	(19)
	G6PD	HCC Tumor cells	Accelerated glycolysis/Pentose phosphate pathway	Forms dense Metabolic Islands, causing spatial exclusion of CD8+ T cells	Marked immunosuppression; metabolic exhaustion **Evidence:** HCC spatial metabolic core; etiology NR; SA.	(74)
	TDO2	HCC Tumor cells	Tryptophan depletion/Kynurenine production	Acts as a metabolic disruptor; impedes TLS maturation to form Aberrant TLS	Disrupts local immune command centers **Evidence:** HCC TLS/metabolic studies; SA.	(38)
	Aberrant TLS	FOLR2+ Tregs/M2-like TAMs	Suppressive infiltration into lymphoid aggregates	Create dysfunctional regulatory hubs; drive local T cells into terminal exhaustion	Fails to restrict tumor progression **Evidence:** HCC TLS studies; SA.	(38, 39)
	FAP+ CAFs	Stromal cells	TGF-β, PDGF, and ADM signaling	Physically entangle and impair infiltrating PD-1+ CD8+ T cells	Marked core immunosuppression **Evidence:** HCC CAF studies; SA+functional inference.	(44, 56, 58)
	M2-P3 TAMs	Tri-phosphorylated TAMs	Terminal phosphorylation	Form a synapse of suppression with hepatocytes by upregulating PD-L1/CD47	Associated with neighboring T-cell dysfunction; pronounced immune suppression **Evidence:** HCC spatial proteomics; SA.	(42)
	DAB2+ TAMs	Macrophages	Crosstalk with FAP+ CAFs	Establish hostile, immunosuppressive macrophage-fibroblast hubs	Poor clinical outcome **Evidence:** HCC macrophage-CAF studies; SA.	(58)
	CCL15 & CD163	Core Macrophages	Spatial co-expression	Marks a trajectory of advanced immune dysfunction within the deep core	Unfavorable prognosis **Evidence:** HCC tumor-core macrophage studies; SA.	(40)
	CCR7	HCC Tumor cells	AKT/ERK pathway activation	Drives malignant aggression and invasive pseudopodia	Accelerated tumor progression **Evidence:** HCC tumor-cell studies; CMI/FV association.	(69)
	PRKDC & HMGB2	HCC Tumor cells	Error-prone DNA repair; niche programming	Create genomic instability hotspots and drivers of metastatic niches	High risk of distant metastasis; rapid tumor evolution **Evidence:** HCC malignant-clone studies; SA.	(73)
	NDRG1	NDRG1+ TAMs	STAT3/NF-κB pathway	Establishes localized checkpoints; spatially excludes effector CD8+ T cells	Unfavorable clinical prognosis **Evidence:** HCC scRNA-seq/spatial studies; CMI+CA.	(76)
	PROM1+ CSCs	Cancer Stem Cells	Spatial overlap with Treg clusters and CD47+ cells	Create a protected niche shielded from immune attack	Maintenance of the CSC pool; tumor recurrence **Evidence:** HCC CSC spatial studies; SA.	(48)
Vascular, PVTT & MVI Niche	ODC1	Tumor/PVTT tissues	Polyamine synthesis	Creates a polyamine shield via nitrogen metabolism control	Drives immune evasion in portal vein tumor thrombus **Evidence:** HCC-PVTT; public bulk cohorts plus spatial/mIHC validation; CMI+CA.	(64)
	LAMP3 & TREM2	LAMP3+ DCs & TREM2+ TAMs	Synergistic interaction with cycling T cells	Define microvascular invasion regions as distinct biological metastatic niches.	High mortality and systemic dissemination **Evidence:** HCC MVI/PVTT-region studies; SA.	(35)
	SORT1 & GOLM1	Core Tumor Subclones	Oncogene signaling; VEGFA/CXCL5	Promote subclone-specific angiogenesis; physically co-localize with EC1/EC5 subsets	Provide oxygen and routes for aggressive dissemination **Evidence:** HCC subclone/vascular niche studies; SA.	(33, 34)
	CD63 & UQCRH	Endothelial Cells (via exosomes)	Exosome release; ROS accumulation	Induce global immunosuppression; inhibit antigen presentation	Vascular-mediated immune evasion **Evidence:** HCC endothelial/exosome studies; functional inference; sample/etiology NR.	(30, 75)

pTLS, peritumoral tertiary lymphoid structure; SA, spatial association; CMI, cross-modal inference; FV, functional validation; CA, clinical association. The evidence context entry lists HCC cohort, sample type, etiology, or functional model information when available; NR indicates that the cited summary did not uniformly report this detail. A reported feature may satisfy more than one evidence category.

Complementary functional models provide a practical bridge between spatial discovery and treatment prediction. Recent HCC patient-derived organotypic tumor spheroids (PDOTS) derived from 30 patients preserved genomic, immune, and histopathological features and demonstrated immune-dependent responses to PD-1 blockade; *ex vivo* response showed 80% concordance with matched patient-derived xenograft models, and the derived Organoid Killing Index correlated with outcome in an external atezolizumab-bevacizumab cohort (*r* = 0.829, *P* < 0.001) (78). PDOTS is not itself a spatial assay and therefore does not prove that a given spatial association is causal. Rather, it offers a biologically relevant system in which spatially defined candidate states can be prospectively tested for regimen-specific functional sensitivity.

### 4.2 Spatially weighted prognostic models

The spatial organization of tumor ecosystems can strongly influence patient outcome. A spatial competition pattern, observed in approximately 30% of cases, is associated with high ITH and poorer prognosis than monolithic tumors (79, 80). Immune gradients provide additional prognostic information. A core-suppressed pattern defined by CCL15 and CD163 co-expression in the deep tumor core represents a trajectory of immune failure, whereas an immune-enriched pattern marked by peritumoral CCL19/CCL21 indicates lymphoid recruitment and favorable outcomes (40). Local cell-cell proximity also matters: closer tumor-CD8+ T-cell interactions were associated with improved overall survival in a discovery cohort of 68 HCC biopsies and an independent validation cohort of 190 biopsies (81). Dense barrier structures around the tumor core are similarly associated with metastasis risk and ICI non-response (55, 60).

Spatial prognostic models should report the discovery and validation cohorts, clinical setting, endpoint, effect estimate, and calibration/performance measures, rather than a spatial association alone. One example is the Tumor-Immune Microenvironment Spatial Heterogeneity (TIMES) score, a spatially weighted recurrence model developed from spatial immune profiling of 61 HCC patients and validated in 231 patients from five multicenter cohorts (82). The original report describes a large recurrence-risk association (reported HR = 88.2, P < 0.001), together with real-world accuracy of 82.2% and specificity of 85.7%. However, the main text and accompanying supplementary/source-data files do not report a 95% confidence interval for this HR. Given the unusually large effect estimate, this HR should be interpreted as model- and endpoint-specific rather than as a broadly generalizable clinical hazard estimate (82). Metabolic features can also be incorporated, but their statistical context should be stated explicitly. In PVTT-focused spatial multi-omics, Fib-C3, T-C3, and T-C7 metabolic meta-clusters contributed to recurrence-free survival, and Fib-C3 showed a negative association with overall survival. A combined PVTT-enriched metabolic index predicted PVTT occurrence (*AUC* = 0.722, 95% CI 0.580-0.864), overall and recurrence-free survival in The Cancer Genome Atlas Liver Hepatocellular Carcinoma (TCGA-LIHC) (*P* < 0.001), and immunotherapy response (*AUC* = 0.822, 95% CI 0.599-1.000) (64). The original PVTT study did not report median survival differences for these metabolic meta-clusters; therefore, these model-performance and survival-association statistics should be distinguished from direct survival-time estimates.

### 4.3 Reprogramming the architecture for immunotherapy

A clinically useful spatial-engineering strategy should link a defined barrier, a perturbable mechanism, and a regimen-specific endpoint. Locoregional therapies provide one route: transarterial chemoembolization (TACE) can alter the spatial relationship between CD8+ T cells and Tregs in necrotic regions, whereas hepatic arterial infusion chemotherapy (HAIC)-associated spatial profiling has implicated TLS formation and redistribution of intermediate-exhausted GZMK+ PD-1+ CD8+ T cells (83, 84). These observations motivate, but do not by themselves prove, a causal treatment mechanism.

Physical and metabolic barriers remain a major constraint on therapeutic efficacy. Activated HSCs and α-SMA+ CAFs can construct protected niches reinforced by DDR1 and TGF-β signaling, which are associated with reduced lymphocyte access (56, 85). Macrophage subsets can further sequester T cells through ARG1 and ROS production (86). Treg expansion, supported by neutrophil extracellular traps (NETs) and IL-17, adds another suppressive layer (87, 88). Because these barriers differ in how directly they have been tested, they should not be given the same therapeutic weight. Spatial mapping can nominate DDR1/TGF-β, ARG1/ROS, NET/IL-17, or CAF-macrophage niches as candidate targets; stronger functional support requires blockade, genetic perturbation, coculture, or *in vivo* validation before a niche is framed as a therapeutic mechanism.

Targeted and anti-angiogenic therapy provides a complementary route to spatial immune remodeling. In HCC, anlotinib enhanced anti-PD-1 activity in preclinical models and was linked to suppression of TFRC through the VEGFR2/AKT/HIF-1α pathway, increased CXCL14 expression, and greater CD8+ T-cell infiltration (89). This study directly connects a perturbable tumor-cell pathway with chemokine remodeling, T-cell access, and checkpoint-blockade efficacy. Recent HCC data also provide functional support for an FGF-related immunoregulatory strategy: in an anti-PD-1-resistant HCC model, FGFR1 upregulation activated MAPK signaling and induced SPP1 expression; SPP1 knockdown or neutralization reduced macrophage accumulation and restored intratumoral T-cell infiltration. Pharmacological FGFR1 inhibition with BGJ398 synergized with anti-PD-1 therapy in preclinical models (90). These findings support FGFR1-SPP1 as a candidate route to reprogram an immunosuppressive microenvironment, although prospective clinical validation and direct measurement of stromal architecture remain necessary.

Taken together, the translational sequence is not simply “map, then treat”. It is “identify a spatial state, test its function in a perturbation-capable HCC model, and then assess its predictive value in a regimen-defined clinical cohort”. Evidence should therefore be reported separately for anti-PD-1 monotherapy, atezolizumab plus bevacizumab, dual checkpoint blockade, and TACE/HAIC combinations, rather than as a single undifferentiated category of immunotherapy. At present, spatial biomarker-to-regimen mapping remains uneven. HCC evidence is strongest for anti-PD-1-containing regimens, atezolizumab-bevacizumab, and HAIC/TACE-linked contexts. Mortality, MVI/PVTT, recurrence, and survival associations should be reported using the original endpoints and statistics rather than as regimen-agnostic predictors.

## 5 Discussion

The integration of single-cell transcriptomics with spatial omics has changed how HCC biology is interpreted. Spatial technologies provide the tissue context that dissociative scRNA-seq lacks. Whereas scRNA-seq catalogs cellular identities, spatial coordinates show how those identities are arranged and how local neighborhoods shape immune function. In this view, immunosuppression in HCC is not only a consequence of soluble cytokines or transcriptional exhaustion. It is also a structural process shaped by physical zoning (40, 79). The identification of the TIB (55, 59, 91) supports a proximity-based model in which spatial sequestration contributes to ICI resistance (81).

Expanding beyond transcriptomics to spatial metabolomics, including MALDI-MSI and DESI-MSI, is also important for decoding the TME. Spatial metabolomics can visualize metabolic exclusion barriers that complement stromal barriers. Mapping lipotoxic niches, lactate-PUFA gradients at tumor-CAF interfaces (57), and ammonia-recycling circuits used by Tregs (65) can convert inferred metabolic pathways into spatially resolved maps. These approaches clarify how nutrient deprivation and oncometabolite accumulation may restrict local T-cell function.

Despite these advances, several technical gaps remain. Cross-platform consistency and platform-specific bias are major challenges (92). Current HCC spatial architectures have been defined using platforms with different trade-offs. Sequencing-based methods such as Visium and Stereo-seq provide broad gene coverage but often at multicellular resolution, which can obscure direct cell-cell contacts (93). Imaging-based methods such as MERFISH, Xenium, and CosMx achieve subcellular resolution but are constrained by predesigned gene panels (94). Future studies should test whether key niches, including the TIB and TLS, are reproducibly quantified across platforms.

Clinical translation of spatial biomarkers also faces practical barriers. High cost, complex data integration, the need to combine fresh-frozen spatial metabolomics with formalin-fixed paraffin-embedded transcriptomics, and prolonged turnaround times currently limit routine use in pathology. A near-term goal is to distill high-dimensional spatial information into simplified spatial signatures, such as standardized multiplex immunohistochemistry panels that can fit existing hospital workflows. Recognizing these technical and economic boundaries will be essential for translating the spatial blueprint of HCC into precision immunotherapy.

An additional limitation is cohort composition. Many HCC spatial-omics studies have analyzed resected, treatment-naive, and relatively early-stage tumors, whereas systemic immune checkpoint therapy is commonly used in unresectable or advanced disease. The spatial architectures described in surgical specimens may therefore not fully represent metastatic, post-treatment, or advanced-stage lesions. Prospective studies should include biopsy-based and longitudinal samples from regimen-defined advanced HCC cohorts to determine whether candidate spatial features are conserved and predictive in the population receiving systemic therapy.

Evidence specificity also varies across the mechanisms reviewed here. We prioritize HCC-specific spatial or functional studies when available, and use evidence from precursor liver disease, pan-cancer myeloid biology, or other tumor types only as supportive and hypothesis-generating context. To make this hierarchy explicit, **Table 2** now reports the HCC evidence context and evidence level for key spatial biomarkers where these details are available.
